# Where is the patient in models of patient-centred care: a grounded theory study of total joint replacement patients

**DOI:** 10.1186/1472-6963-13-531

**Published:** 2013-12-23

**Authors:** Fiona Webster, Anthony V Perruccio, Richard Jenkinson, Susan Jaglal, Emil Schemitsch, James P Waddell, Samantha Bremner, Melanie Hammond Mobilio, Viji Venkataramanan, Aileen M Davis

**Affiliations:** 1 Department of Family and Community Medicine, University of Toronto, 500 University Avenue, 5th Floor, Toronto, Ontario M5G 1V7, Canada; 2 Arthritis Program, Division of Orthopaedic Surgery, University Health Network and Institute of Health Policy, Management & Evaluation, 399 Bathurst Street, EW1-449, Toronto, Ontario M5T 2S8, Canada; 3 Division of Orthopaedic Surgery, Sunnybrook Health Sciences Centre, 2075 Bayview Avenue, MG 361, Toronto, Ontario M4N 3M5, Canada; 4 Department of Physical Therapy, University of Toronto, 160-500 University Avenue, Toronto, Ontario M5G 1V7, Canada; 5 Division of Orthopaedic Surgery, St. Michael’s Hospital, 55 Queen Street East, Suite 800, Toronto, Ontario M5C 1R6, Canada; 6 Division of Orthopaedic Surgery, St. Michael’s Hospital, 38 Shuter Street, 3rd floor, Room 3005, Toronto, Ontario M5B 1A6, Canada; 7 Faculty of Medicine, McMaster University, 1280 Main Street West, Hamilton, Ontario L8S 4L8, Canada; 8 Division of Health Care and Outcomes Research, Toronto Western Research Institute, University Health Network, 399 Bathurst Street, MP11-324, Toronto, Ontario M5T 2S8, Canada; 9 Division of Health Care and Outcomes Research, Toronto Western Research Institute, University Health Network, 399 Bathurst Street, MP11-322, Toronto, Ontario M5T 2S8, Canada

**Keywords:** Osteoarthritis, Hip and knee replacement, Models of care, Qualitative methods, Patient experiences of care, Patient-centered care

## Abstract

**Background:**

Patient-centered care ideally considers patient preferences, values and needs. However, it is unclear if policies such as wait time strategies for hip and knee replacement surgery (TJR) are patient-centred as they focus on an isolated episode of care. This paper describes the accounts of people scheduled to undergo TJR, focusing on their experience of (OA) as a chronic disease that has considerable impact on their everyday lives.

**Methods:**

Semi-structured qualitative interviews were conducted with participants scheduled to undergo TJR who were recruited from the practices of two orthopaedic surgeons. We first used maximum variation and then theoretical sampling based on age, sex and joint replaced. 33 participants (age 38-79 years; 17 female) were included in the analysis. 20 were scheduled for hip replacement and 13 for knee replacement. A constructivist approach to grounded theory guided sampling, data collection and analysis.

**Results:**

While a specific hip or knee was the target for surgery, individuals experienced multiple-joint symptoms and comorbidities. Management of their health and daily lives was impacted by these combined experiences. Over time, they struggled to manage symptoms with varying degrees of access to and acceptance of pain medication, which was a source of constant concern. This was a multi-faceted issue with physicians reluctant to prescribe and many patients reluctant to take prescription pain medications due to their side effects.

**Conclusions:**

For patients, TJR surgery is an acute intervention in the experience of chronic disease, OA and other comorbidities. While policy has focused on wait time as patient/surgeon decision for surgery to surgery date, the patient’s experience does not begin or end with surgery as they struggle to manage their pain. Our findings suggest that further work is needed to align the medical treatment of OA with the current policy emphasis on patient-centeredness. Patient-centred care may require a paradigm shift that is not always evident in current policy and strategies.

## Background

Access to hip and knee replacement, specifically reduction of wait times for these procedures, has been a concern of publically funded health care systems for the past decade or more. The UK and Canada are but two examples where health systems policies and initiatives have been implemented with attention to access, quality and cost containment [1,2]. While the policies and system responses in terms of implementation have been tailored to country and regional contexts, there are commonalities in the policy approach (e.g. defined benchmarks for wait times from surgical consultation to surgery date) and in aspects of restructuring of care provision. For example, the UK and some jurisdictions in Canada have focused on improving access through implementation of a model of care that allows triage to the surgeon for those who are deemed potential surgical candidates [3-8]. Other models have incorporated case managers or processes to aid the patient in navigating the system and care pathway once there has been a decision for joint replacement [9]. In Canada, there is a general sense that the identification of reduced wait times for hip and knee replacement surgery as a priority in the 2004 Federal Health Accord, and the implementation of strategies to improve access to care, have resulted in reduced wait times. Yet challenges remain in achieving the benchmark wait time of 26 weeks from surgeon/patient decision to surgery date [2]. What is not clear is whether these initiatives, implemented in response to policy, are patient-centered in terms of responding to ‘individual patient’s preferences, needs and values’ [10]. In other words, is the wait time for surgery and even surgery itself the defining moment in the patient’s experience of osteoarthritis (OA)?

The concept of patient-centred care is a term that has permeated the literature and government policy since the 1990s [11]. The Institute of Medicine, National Academies of Science (US) defined patient-centred care as care that is ‘respectful of and responsive to individual patient preferences, needs, and values’ with clinical decisions guided by patient values [10]. Despite the seemingly self-explanatory nature of the term, it is in fact poorly described and variably used [11-15]. Additionally, fiscal realities have resulted in government policies that emphasize cost containment through efficiencies that improve or maintain access and quality care. Whether or not such policies are or can be patient-centred remains unexplored.

Most studies have explored the experiences of people with OA in relation to their referral for consideration of hip or knee replacement rather than solely on the impact of OA on their lives. Existing literature focuses on primary care physicians’ challenges in managing musculoskeletal conditions [16], tools to define who is a candidate for hip or knee replacement [17] or shared decision-making about proceeding to surgery [18,19]. Literature based on the patient experience has often been gathered through patient satisfaction surveys evaluating the process of care [20]. However, there have been multiple criticisms of these surveys [21,22] including but not limited to: issues with reliability and validity [23]; patients’ lack of understanding of the processes of care [24,25]; and, the tendency for patients, especially elderly patients, to express positive satisfaction despite receiving poor care [26,27]. Much excellent qualitative research has focused on specific aspects of the patient’s experience; for example, multiple issues related to pain and pain management [28-30]; medication adherence [31]; patient education needs [32]; and treatment decision-making including decision to have TJR surgery [29,33-35]. However, none of these studies have been explored in relation to policies set at the systems level that in fact shape the patient experience of their OA.

Given this gap, this study sought to elicit and document the experiences of people scheduled to undergo total hip or knee replacement surgery, focusing on the impact of OA on their lives rather than on their decision-making or referral wait times to having TJR. We discuss how patients’ experiences of OA pose a challenge to the rhetoric of patient-centred care, based on the definition of the Institute of Medicine, National Academies of Science (US) [10] and suggest how these challenges can be addressed. Our study draws on the idea that language is active; that is, it has a constitutive function as well as a descriptive one [36]. Rather than simply providing labels for things, words establish the common understandings that set up how we can think about things in any given situation. Arguably, the notion of “patient centredness” has become ubiquitous internationally and our study sought to explore how several policies related to TJR may or may not align with the experiences and needs of patients with OA.

## Methods

### Study design

This research is part of an ongoing three year qualitative study with the primary purpose of investigating why people with OA do or do not engage in social and personal roles and instrumental activities of daily living after having a primary TJR. Each participant will be invited to take part in three interviews: (1) approximately one month prior to surgery; (2) nine months post-surgery; and, (3) 18 months post-surgery. For the purposes of this paper, only data from the preoperative interviews are used in analysis. A constructivist approach to grounded theory which guided sampling, data collection and analysis was adopted [37]. This approach theorizes that people assign subjective meaning to their everyday experiences and was selected in light of the limited information available on the patient experience of the primary objective under study. Participants were recruited from the practices of two orthopaedic surgeons using first maximum variation and then theoretical sampling based on age, sex and joint replaced (hip or knee) [38]. The qualitative sampling was purposive and required that sufficient data were generated to sufficiently explore the issues under investigation. The sampling strategy was designed to a) explore potential differences; b) strategically allow a more theoretically stratified approach to data collection at a later stage in the study dependent upon findings that might be identified. For example, the existing literature suggests that age and sex might play an important role in patient experience and we therefore established these parameters as categories to ensure robust exploration of these factors.

The data reached a point of saturation when no new information or themes were being generated, at which point interviewing stopped [39]. Data saturation of course is a sometimes nebulous concept in the work of qualitative researchers as there are few clear guidelines beyond individual researcher experience and judgment for establishing when saturation has been achieved [40,41]. Our team collaboratively determined that saturation had been achieved through extensive team meetings and transcript review. Ethics approval was obtained from the concerned institutional review boards prior to commencing the study and each participant provided informed written consent.

### Data collection

Potential participants were identified from the surgeons’ roster data and mailed an information letter describing the study and a consent form. A research associate followed up by telephone to answer any questions, screen for eligibility and arrange an interview time for interested participants. Patients were eligible for participation if they received a diagnosis of OA and were fluent in English; patients with rheumatoid arthritis or other illness that might limit activity and participation (e.g. Parkinson’s, multiple sclerosis, etc.) were excluded. A semi-structured interview guide facilitated discussions with participants. The guide was piloted to determine if length and flow of questioning was appropriate prior to commencing the study. All questions were meant to be exploratory to allow differences between patients’ perceptions and experiences to emerge during the course of the interview. Preoperative interviews occurred about 1 month prior to surgery from November 2011 until July 2012 and took place in person in the participant’s home or a private room in the hospital or by telephone. The literature supports the use of both telephone and face-to-face interviews within the same study without compromising the trustworthiness of the findings [42]. All preoperative interviews were conducted in English by an interviewer experienced in qualitative research. Interviews ranged in length from 12 to 48 minutes. In total, 33 participants (17 female, 16 male) were included in this analysis. The age of participants ranged from 38 to 79 years, and 20 were having their hip replaced while 13 their knee replaced (Table 1).

**Table 1:** Description of the participants

**Age group**	**HIP**		**KNEE**		**Total**
	**Male**	**Female**	**Male**	**Female**	
30-45	3	1	1		**5**
46-65	6	4	2	4	**16**
66-80	2	4	2	4	**12**
Sub-total	11	9	5	8	
**Total**	**20**		**13**		**33**

### Data analysis

Each interview was digitally recorded and transcribed verbatim. Transcripts were imported into NVivo, a qualitative software program that helps organize and retrieve data [43]. Four researchers each independently coded a subset of transcripts, met to discuss their interpretations and developed a coding framework. This group consisted of the two lead investigators, the interviewer and a research assistant (FW, AD, SB, VV). The RA then coded the remaining transcripts using NVivo 9 software for data management. Through an ongoing series of meetings an iterative process was undertaken in which new transcripts were read and the coding framework evolved, with careful attention to the use of language in both participants’ accounts and how our codes were named. The regular meetings also provided an opportunity for reflexive sharing as the research team considered how their assumptions and beliefs might impact interpretation of data. Codes were then organized into categories and themes were constructed. Simultaneous data collection and analysis allowed for emerging themes to be pursued in subsequent interviews in keeping with the underlying tenets of grounded theory. Like most qualitative analysis methods, grounded theory is based on the concept of emergent themes. These themes are not just used to explore an issue, but also to construct a cohesive idea or theory about an investigated phenomenon. The present analysis followed the constructivist approach to grounded theory described by Charmaz [37] emphasizing connections between theory, concepts and empirical data [44]. Theory enters a research study at different points [45] and in our study we adopted a critical theoretical lens that drew on aspects of discourse analysis and linked people’s experiences back to the social organization of their care. We did not attempt to establish the ‘truth’ of participants’ accounts measured against an objective reality but sought to understand the meaning they assigned to their experiences. An audit trail was maintained to ensure the trustworthiness of the analysis. Summaries were written after each interview and memos were created after each research team meeting. The RATS guideline for reporting qualitative research was used to ensure quality in the reporting of our study in relation to sampling, recruitment, role of researchers, ethics, analysis and discussion [46].

## Results

We have organized our findings around four main themes that emerged from patient experiences with care and which highlight potential challenges for existing care which need to be redressed. These are: 1) patients’ experience of multiple joint symptoms and multiple chronic conditions; 2) the experience of OA as a chronic disease; 3) concerns related to prescription pain management; and, 4) referral times to surgical consultation for consideration of TJR. These findings suggest that patients’ descriptions of their OA as a complex and chronic condition challenges the doctrine of patient-centred care delivery models that largely focus on wait times for surgical interventions.

### The experience of multiple joint symptoms and multi-morbidity

Patients described having multiple painful joints and other health conditions that both impacted and were affected by their OA. Many described this as progressive, with their OA pain being worse in one joint and then eventually becoming worse elsewhere:

*The right side is worse than the left side, but lately, for the past four or five weeks, I’ve been feeling the left side also being almost as equally sore as the right side. It’s like somebody has a knife in it and is twisting it.* (P5, male, early 50s)

Painful joints included the opposite side knee and hip, as well as elbows, back, and toes. Involvement of multiple joints exacerbated patients’ pain experience:

*At this point the pain in my hip… you could cut the whole thing off right now from [hip] on down, because it just feels like it’s been thumped with baseball bats right from my ankles all the way up and in, and that’s how much it hurts*. (P25, male, early 60s)

The participants in our study frequently revealed that other health conditions both impacted and were impacted by their OA. In some instances these health conditions were believed to have developed as a result of OA, but they were always described as related, as in the following account:

*I’d developed coronary problems and they would send me to rehab, and I will go there and they will make me walk and will make me do the bike and that, and then I’d be in excruciating pain. For three days, I’d be in bed because my hips would hurt so much*. (P5, male, early 50s)

Participants also described having a variety of other health conditions, including high blood pressure, cardiovascular disease, diabetes, chronic obstructive pulmonary disease, asthma, varicose veins, and acid reflux. The experience of these additional conditions was often described as highly influential on the participants’ quality of life. Due to their complex health needs, participants reported that they were sometimes given contradictory advice by different physicians, as described in the following example:

*… the frustrating thing is one doctor is telling you, you have to lose weight and the next [specialist is] saying don’t move, you have to sit with your leg up. Well, who do you listen to? It’s frustrating.* (P33, female, late 40s)

### The experience of OA as a chronic disease that impacts on physical and social functioning

For the participants in our study, OA was described as a chronic, life-changing condition that had an impact on every aspect of their lives, from the moment they woke up to the time they went to bed. Participants described struggling with sleep because of their OA pain. Many patients also reported feeling fatigued; often, sleeplessness and fatigue were described by participants as being linked. Fatigue has been recognized as a serious and disabling symptom of several diseases, and recent work suggests this is the case with OA as well [47]. One participant shared her frustrations with her lack of sleep in detail:

*There’s been maybe three nights where I’ve just about been in tears. It’s like I’ve tried everything I can try, how come it won’t go away, but fortunately only three [nights I haven’t slept].* (P13, female, mid 60s)

Participants described how OA pain negatively impacted their employment and several described having to reduce their duties at work to accommodate OA-related limitations. For many, OA pain also required the modification of duties on the job, or the elimination of some responsibilities entirely. Participants expressed a desire to continue to work, and would modify their physical activities by being “careful”, reducing hours, or eliminating duties as necessary.

*It just was very difficult to get through the work day. I mean, I was put on modified duties for such a long period of time because, well, my surgeon at the time… didn’t feel that I could ever return to regular duty … because of my knees being so bad….* (P11, female, late 40s)

Participants described feeling as though their OA was negatively impacting the size of their social sphere. People who previously engaged in regular social activities outside the home, such as going to the movies, skiing, etc., were unable to continue these activities in the same ways they once had. For many, the types of outings they used to enjoy became increasingly difficult as the pain of OA progressed, resulting in less time spent outside the home. Consequentially, participants’ social circles became increasingly limited, as their more home-centred life meant less interaction with those outside their immediate surroundings.

Participants described the impact of their OA pain as permeating beyond their participation in everyday activities and into their relationships with family members. For some, the necessity to reduce or eliminate physical activities meant more than simply an inability to do the activity; it meant a change in the ways in which they could interact with loved ones. For participants whose marriages had regularly included activities such as dancing, having to give up this interaction with their spouse was particularly difficult. For others, the struggle with the constancy of OA pain rendered the maintenance of normal relationships with family members difficult. Participants who had previously been cheerful and happy people began to feel as though they needed to filter their daily interactions with family members in order to prevent the experience of pain from negatively impacting their interaction. This was described by one participant as the need to “think before I speak” instead of it “just coming out” (P13, female, mid 60s).

Overall we heard in participants’ accounts a strong sense that their lives were shrinking.

*My ability to do extracurricular things like my walking distance has shrunk. I can’t cross-country ski anymore. It’s just too painful and snowshoeing I can’t do. And sitting for long periods of time, it becomes very uncomfortable. I had to give up part of my job… everything seemed to shrink, what I could do and couldn’t do.* (P6, female, early 50s)

*Strangely enough, I understand how old people feel, how when you get to a certain age you start to narrow your social field and what used to be here at 20 is now here at 80, but for me I’m here at 40. You really do sense every option has a yes and a no and more often, the no is dictated by the amount of pain you want to feel, the amount of pain you know you are going to feel and whether or not you want to go through it. [OA] really does narrow your social circle.* (P32, male, mid 40s)

### Patient concerns with prescription pain management

Patients described their difficulties in obtaining adequate pain relief for their OA pain, as well as their own concerns about prescription pain management. Many patients were uncomfortable taking pain medication. The following sentiment was shared by many, *“I don’t like medication so I try to stay away from it. I try to manage [my OA pain] with as few pain killers as possible.”* (P33, female, late 40s) There were many accounts of patients who were told by their primary care providers that they could not have prescription pain medication. As one participant described:

*He said, well take Tylenol for arthritis. I didn’t know you had anything else for [the pain], I just figured that was it. But then when I went one day I was nearly crying and I said, you’ll have to give me something for this.* (P1, female, early 70s)

Participants described trying different medications for pain relief but finding them ineffective: “*… well they want you to try Advil and Tylenol and Aleve and all that before they give you the Celebrex. That’s what the doctor told me, and of course none of them really did too much*.” (P9, female, mid 60s) The issue of pain management was complicated for participants. While they did not want to be in pain, at the same time they also had concerns around addiction and were often resistant to taking pain medication. The following example typifies this tension that was evident in several patient accounts:

*I’d been to [my doctor] a few times and told him I was in a lot of pain and he just said, just take the Tylenol. He only gave me Tylenol …. No that’s not true…he gave me … OxyContin or something, … And I took them and they worked. He only gave me maybe 15 or 20, I don’t remember how many he gave me … Anyway I go back to get more and he went, are you finished these? I went, yeah you gave me them. He said to me, they’re highly addictive. I said, yeah but they work. He said, no we can’t give you that so he gave me something else but it didn’t work*. (P1, female, early 70s).

### Delayed referral times for surgery consultation

It was not surprising that although TJR was not the central focus of their concerns some patients did not want to wait any longer for their TJR surgery. In particular, younger patients described how their physicians thought they should wait for referral to surgery, while they were instead more concerned with their current quality of life than with the risk of future revision surgery:

*[I used to think that] Maybe I’ll have a hip replacement 10 or 15 years from now. I didn’t think I would be here four years from now doing it. But, you know, it gets to the point where you’ve got to do something because you’re giving up doing too many other things.* (P32, male, mid 40s)

In the following account, the physician’s sense that the patient was not yet “experiencing enough discomfort” sharply contradicted her pain experience:

*I was quite disappointed [that I would have to wait for surgery] because I felt like I was having a fair bit of discomfort especially in my left leg. I was already taking prescribed anti-inflammatories and I had a prescription for Tylenol 3 to take PRN so yeah, I was disappointed that they weren’t going to be doing anything for quite a while. And I thought well maybe I’m just not experiencing enough discomfort and I had a fair ways to go. It kind of panicked me a little bit because I was working, still am, and I didn’t know how I was going to get through the next three, four or five years with the pain.* (P6, female, early 50s)

## Discussion

Patients’ accounts of OA suggest that their experiences often involve the simultaneous existence of both multi-morbidity and pain in multiple joints. This does not align with the current policy emphasis on a single joint and access to surgery to replace that single joint, which has been adopted in many jurisdictions [1,2]. Patients emphasized the chronicity of their OA experience as a daily condition that permeated every moment of their day. They described the myriad of ways in which their OA affected their daily lives, their relationships with others, their employment experiences and their other disease conditions and/or joints. From the moment they woke up in the morning to the end of the day when they struggled with sleep, the pain and stiffness experienced was a result of their OA as a constant companion for them. This is in stark contrast to the health system’s perspective of OA, where OA is not currently designated as a chronic disease from a policy and remuneration perspective by various provincial ministries of health in Canada. Although from a systems perspective TJR is classified as a single event, localized to a single diseased joint, from the patient perspective it is one episode in the context of their experience of OA as a chronic disease that impacts multiple joints and multiple aspects of life over a lifetime. Based on their accounts, we argue that TJR is an acute intervention in the context of a chronic disease.

When discussing the time leading up to surgery, many participants described using non-prescription pain or non-steroidal anti-inflammatory medications (NSAIDs), although the efficacy of such medication was described as variable. It was common for participants to feel that their doctor was not comfortable prescribing medication for pain management, instead preferring that the patient take over-the-counter remedies. While patients in our study expressed being fearful of addiction, this limits the pain reduction strategies available to them. The current controversy over the use of opioids for pain management likely underpins the prescribing reluctance of physicians (and patient fear). There is a rising concern with addiction amongst physicians [48] with some referring to this as an epidemic [49] and the fear of potential investigation and/or sanctions against the physician may perpetuate inadequate treatment of pain and negatively influence physicians’ prescribing practices [50]. Also, the controversy stems from concerns around addiction rather than pain management [51]. Some authors have argued that the field of pain medication has been dominated by the scientific domains rather than the social science disciplines and have even argued that there is little scientific evidence for fear of addiction to opiates [51]. For those patients whose pain is not under control, they are sometimes caught between physicians who are reluctant to prescribe prescription medication and the lack of other effective strategies.

The patients in our study often reported having a different assessment of readiness than their physicians who might encourage them to delay referral; this was especially true for those patients who were younger. Rotstein and Alter [52] take issue with the current system of measurement which defines the wait time as the period from surgical consultation to surgery and does not consider delays in the primary care physician initiating a referral or the wait from primary care referral to surgical consult, arguing that there is a problem in when the wait time is thought to begin. This argument resonated with the feelings expressed by our participants, some of whom described enduring long delays before being referred to a specialist. This suggests a need to re-evaluate when wait times really began for those awaiting hip and knee replacement and to increase the support available to patients while they wait given the extraordinary impact of OA on their day to day functioning and quality of life.

Our findings suggest that health policies and strategies (such as the wait time strategy) cannot be considered patient-centred when they are not based in or representative of patient experiences. Developing models of care that are truly patient-centred may require a paradigm shift in how we conceptualize and deliver patient care for OA. Increasingly, the notion of shared decision-making is coming into use as a model for understanding and improving the role of the patient in clinical decision-making [53]. Yet some have argued that the extent to which patients engage meaningfully in conversations about their medical care is not well understood [26] and that clinicians and patients may have differing opinions in relation to priorities and treatment [54]. For example, Brody has argued that the traditional model of patient-provider relationship places patients in a passive role that does not empower them to engage in clinical decision-making. For the OA patient, we argue that it is urgent that future work on the development of care models and approaches to patient management expand to take into account patient experiences as a whole, rather than focusing on an acute episode and affected joint as a single entity that is divorced from the lived experience of the person. Access to and processes of care and patient management need to consider the patient’s experiences of multiple symptomatic joints, comorbidities and challenges of day-to-day life in the context of their preferences, needs and values. Additionally, better coordination between primary care and specialist care needs to be established to allow referrals to surgery to be based on agreed upon standards and also to include some patient’s preferences for better quality of life now even with the risk of future revision surgery. This is especially true in the case of younger OA patients.

### Limitations

As with any qualitative study, our findings are based on the experiences of participants. Our participants received their replacement surgery in a large academic hospital in an urban area in Canada. We do not know the extent to which our participant’s experiences are generalizable beyond this setting. Additionally, our analysis did not include patients being treated for depression and/or who were identified as having addiction problems. These patients may have valuable insights to share regarding the analysis we have developed.

## Conclusions

Despite the fact that initiatives have achieved the goal of reducing wait times for hip and knee replacement surgery, the extent to which this goal and or its measurement truly aligns with the needs of OA patients remains unclear. Our findings suggest that further work needs to be done in order for the management of OA–of which TJR is an acute intervention in the context of the chronic pain and disability of OA– to align with the current policy emphasis on patient-centredness. We would argue that policy makers and clinicians need to be aware of and acknowledge the everyday work patients perform in struggling to manage their OA pain and/or mobility limitations in the context of multi-morbidity and of the significant impact of OA on their lives. This would facilitate formal designation of OA as a chronic disease and help create policy that would provide truly patient-centred clinical care and self-management supports with the potential to limit the often unrelenting impact of OA on people’s lives.

## Competing interests

The author(s) declare that they have no competing interests. This work was supported by an unrestricted operating grant from The Arthritis Society (Canada).

## Authors’ contributions

Conceptualization of the project: AD, FW, AP, RJ, SJ, ES, JW Acquisition of funding: AD and FW Recruitment and data collection: VV, SB, MM Data analysis and interpretation: AD, FW, AP, RJ, SJ, ES, JW, VV, SB, MM Preparation of the final manuscript: FW, AD, VV, MM Approval of the final manuscript: AD, FW, AP, RJ, SJ, ES, JW, VV, SB, MM. All authors read and approved the final manuscript.

## Pre-publication history

The pre-publication history for this paper can be accessed here:

http://www.biomedcentral.com/1472-6963/13/531/prepub
